# Exploring Factors That Could Potentially Have Affected the First 1000 Days of Absent Learners in South Africa: A Qualitative Study

**DOI:** 10.3390/ijerph18052768

**Published:** 2021-03-09

**Authors:** Carien van Zyl, Carlien van Wyk

**Affiliations:** Centre for Child, Youth and Family Studies, COMPRES, Faculty of Health Sciences, North-West University, Potchefstroom 2531, South Africa; carienshalom@gmail.com

**Keywords:** absent learners, biological mothers, first 1000 days, Foundation phase

## Abstract

Background: The first 1000 days of life—from conception to the second birthday of children —is widely recognized as the most crucial development phase, which could have long lasting effects on the health and well-being of children throughout their lives. Purpose: The purpose of this study was to qualitatively explore and describe factors that could potentially have affected the first 1000 days of absent learners in the Foundation Phase within the Paarl-East community in the Western Cape of South Africa. Methods: The data for this qualitative descriptive study were collected through semi-structured interviews with 18 biological mothers of absent learners in the Foundation Phase, who resided in Paarl East. The transcribed texts were analyzed by making use of a thematic data analysis. Results: The findings revealed six predominant themes that played a role during the first 1000 days of the lives of these absent learners. Conclusion: It was concluded from the findings in this study that factors, such as health and nutrition of both the mothers and their children, substance use/abuse during pregnancy, toxic stress, support received by the mothers and their children, attachment, attentive care, and stimulation and play, could have affected the first 1000 days of the absent learners in this study. Since this study did not aim to confirm a correlation between the first 1000 days and absenteeism, but solely to explore factors affecting the first 1000 days, conclusions regarding cause and effect was not possible.

## 1. Introduction

The significance of the first 1000 days as a time-period, is broadly asserted: “The first 1000 days of life—the time spanning roughly between conception and one’s second birthday—is a unique period of opportunity when the foundations of optimum health, growth, and neurodevelopment across the lifespan are established” [1].

The first 1000 days is believed to be the greatest development phase where children’s physical, cognitive, and socio-emotional development will have a lifelong impact, later in their lives [2]. The infancy period, from birth to two years, is characterized by rapid development of the physical and nervous systems, influencing other domains of children’s development. The characteristics infants acquire during this phase are, therefore, fundamental for their lives [3]. The developing brain is particularly vulnerable to prenatal influences during the prenatal period [4]. The brain architecture formed during this critical time-period lays either a strong or weak foundation for the health, learning, and behavior of children later in life [5]. Furthermore, brain development during infancy endures explosive growth, and forms the building blocks of the lives and future of children.

All children must not merely survive but thrive and reach their full potential. It is, therefore, critical to detect risk factors that impact the physical, mental, moral, spiritual, and social development of children [6]. In order for children to reach their full potential, they need nurturing care from birth up until they are two years old, which includes good healthcare, nutrition, security, safety, responsive caregiving, and early learning [7]. The absence of these nurturing care factors during the first 1000 days could result in physical, emotional, and social challenges in the future [8]. A disfavored first 1000 days could, therefore, affect children’s neurological and biological adaptations throughout their lives [9]. 

In South Africa, the Western Cape Government acknowledged the importance of the first 1000 days of life by launching the First 1000 Days campaign, entitled ‘Right Start Bright Future’ in 2016. The aim of this campaign was to raise awareness regarding the importance of the first 1000 days among health workers, other professionals, as well as the general public, by means of workshops and social media. The intention was to further shift the perceptions of healthcare workers and other professionals, to consequently improve health and social services to children during this crucial time-period [10]. This campaign focused on three key areas, namely—(1) health and nutrition; (2) love and attention; and (3) play and stimulation. These three key areas play a fundamental role in the development of children’s physical, social, emotional, and cognitive domains [11]. These distinct key areas respectively involve assorted factors. The first key area, health and nutrition, includes various factors like nutritional deficiency, malnourishment, the physical and mental health of both mothers and their children, and substance use/abuse during pregnancy. The second key area, love and attention, incorporates factors like support to both mothers and their children, nurturing care, and attachment. The last key area, play and stimulation, refers to the stimulation and protection of children [12]. 

The rational for concentrating on ‘absent learners’ was motivated by the author’s work context at the Khula Development Group, a registered non-governmental organization (NGO), rendering services to absent learners and their families. In South Africa, school absenteeism is a huge concern with alarming statistics of 616,327 learners who were absent in the Cape Winelands District, Western Cape, in 2017 [13]. South African policies include various definitions in terms of absenteeism, including, ‘absent’ for any learner not in class or participating in school activities when the class register is marked, as well as ‘continuous absenteeism’ for learners absent from school for ten consecutive days without a valid reason [13]. In this study, the term ‘absent learner’ refers to any form of absenteeism, whether continuous or irregular. There are numerous reasons for school absenteeism, such as poverty, lack of transport, unsuitable housing, children taking care of their siblings, lack of health care, chronic diseases, disabilities, poor nutrition, children’s lack of interest in the curriculum, bullying, lack of family support, working conditions of parents, negative role modeling of parents, parents’ lack of understanding regarding the value of school attendance, parents’ lack of education, as well as problems within the family structure like divorce or domestic violence [14,15,16]. In terms of South Africa, the following reasons for school absenteeism is highlighted, namely poverty, parents’ inability to afford a school uniform or school fees, lack of transport, poor nutrition, child labor, dysfunctional families, gang violence, chronic illnesses of parents or children, learning disabilities, and psychological challenges [13,17]. The multi-level consequences of school absenteeism include poor academic performance, and an increased school drop-out rate with long-term outcomes, such as inadequate education, unemployment, financial instability, and health-related problems [18]. 

The authors were particularly interested in the Foundation Phase, as this is the first phase of formal schooling in South Africa. The Foundation Phase in South Africa refers to Grade R to 3, where learners might enroll for Grade R in the year that they turn six and be admitted to Grade 1 when they turn seven years old. [19]. During this first phase of formal schooling, which establishes the starting point for the academic growth of learners [20], children are expected to master the formal curriculum content, including reading, writing, counting, and calculating [21]. The ability of children to succeed in school is determined by their behavior, social engagement with others, and their capacity to obtain literacy and numeracy skills that are interrelated with their physical, motor, social, emotional, moral, and spiritual development [22]. Children struggle with the curriculum in higher phases if they do not acquire basic reading skills during the Foundation Phase [23]. It is, therefore, of the utmost importance that the early development of children is promoted before they enter the formal school system [24].

From the literature, it is evident that there are many factors transpiring during the first 1000 days that can potentially affect the schooling of children later in life. These factors are—(1) nutritional deficiencies; (2) substance abuse during pregnancy; (3) toxic stress; (4) attachment; and (5) stimulation. Malnourishment, including iron deficiency during the first 1000 days, could lead to reduced cognitive abilities, insufficient school achievement, grade repetition, school absenteeism, and school dropout, later in life [25]. Although substance abuse during pregnancy might involve various substances, such as tobacco, alcohol, Marijuana, or other illicit drugs [26], the effects of alcohol are highlighted. Excessive alcohol abuse during pregnancy could lead to Fetal Alcohol Spectrum Disorder (FASD), a collection of physical, cognitive, and neurobehavioral abnormalities [27,28]. Children with FASD might struggle with learning and behavior challenges at school, such as hyperactivity, memory difficulties, problem solving, social problems, reading, lack of attention, disruptive behavior in class, disobeying school rules, absenteeism, suspension, and school dropout [29,30]. Toxic stress weakens the architecture of the developing brain that could result in long-term learning problems [31]. It is further confirmed that extreme exposure to stress during pregnancy could cause long-lasting emotional and cognitive problems, whereas stressful experiences after birth could alter the neurobiology of children that decreases their ability to succeed in school [32]. Secure attachment in infancy is associated with a positive relationship between children and their school teachers, higher self-esteem, and greater resilience, whilst disorganized attachment during infancy correlates with a series of developmental problems in school, including aggressive behavior, poor relations with peers, and cognitive immaturity [33,34]. Adequate stimulation during infancy results in better cognitive and educational performance later in life [35]. However, learning at school is influenced by the lack of learning, and stimulation in the early years [36].

The purpose of this study was to qualitatively explore and describe factors that could potentially have affected the first 1000 days of absent learners in the Foundation Phase in the Paarl-East community in the Western Cape of South Africa. The authors acknowledge the existence of many variables, besides the first 1000 days, that could influence schooling and absenteeism. For this reason, this paper does not aim to confirm a correlation between the first 1000 days and absenteeism, but solely to explore factors that could potentially have affected the first 1000 days of absent learners in the Foundation Phase.

## 2. Materials and Methods

### 2.1. Research Approach and Design

A qualitative approach was applied to provide insight into the social world of these biological mothers and their children, by producing descriptive data in the participants own words [37,38]. A qualitative descriptive design was utilized for the purpose of this study to obtain a collection of rich and descriptive information, by conducting individual interviews [39,40,41,42].

### 2.2. Research Setting and Sampling

The study was conducted amidst the NGO sector, specifically within the Khula Development Group’s context that renders services to absent learners and their families in the Paarl-East community. The process for service delivery commences when schools in disadvantaged communities refer absent learners to this organization, where they are contacted by the means of home visits. Hereafter, continuous services are rendered, which include home and school visits to provide academic and psychosocial support to absent learners, as well as parental guidance and support to their primary caregivers [43]. The context of the Paarl-East community is characterized by a low-income population, high levels of crime and violence, together with substance abuse [44,45].

A random purposive sampling method was utilized for recruiting 18 biological mothers of the Foundation Phase learners from Grades R to 3, on the database of the Khula Development Group. These learners were referred to the Khula Development Group for a variety of reasons for school absenteeism, such as poverty, social ills like substance abuse, and domestic violence at home, transport challenges, chronic illnesses, poor academic progress, etc. Although the possibility exists that these 18 biological mothers could possibly have older children that was also absent and were recorded on the database of the Khula Development Group, the focus of this study was learners from Grade R to 3. The inclusion criteria were as follows—(1) biological mothers of absent learners in the Foundation Phase; (2) residing in Paarl-East; (3) lived with their child during their child’s first 1000 days; (4) currently lives with their child; and (5) were factually capable of giving consent to participate in this research study. 

### 2.3. Procedure

Ethical approval was obtained from the registered Health Research Ethics Committee at the North-West University (NWU-000008-19-A1), South Africa, which conforms to the principles outlined in the Declaration of Helsinki. Written permission to conduct the study at the Khula Development Group was obtained from the chief operational officer of this organization, who acted as a gatekeeper. The branch manager and two other employees at the Khula Development Group fulfilled the role as mediator and independent persons during the recruitment process. The independent persons conducted home visits to recruit participants, and they were also responsible for obtaining written consent forms from all participants. Arrangements were made to conduct the interviews with the participants at the offices of the Khula Development Group. 

### 2.4. Data Collection

Semi-structured individual interviews were conducted with all of the participants, which lasted approximately one hour each. An interview schedule consisted of 12 questions, along with a timeline tool. The interview schedule covered the three key areas of the Western Cape’s First 1000 Days campaign—(1) health and nutrition; (2) love and attention; and (3) play and stimulation. During the interview, the participants were asked a variety of questions about the time-period of their child’s first 1000 days—from pregnancy until their child’s second birthday. The interview schedule contained two sections, the first section covered questions regarding the pregnancy phase, whereas the second section focused on questions relating to the phase from birth to two years. These open-ended questions were formulated in a simple style, and the following were asked—(1) describe your living conditions while you were pregnant with your child; (2) tell me more about your own health during your pregnancy; (3) explain what you ate and drank during your pregnancy; (4) tell me more about the people who helped and supported you during your pregnancy; (5) describe your feelings towards your unborn baby while you were pregnant with your child; (6) how did you experience the pregnancy?; (7) describe your living conditions from the time your child was born until two years of age; (8) tell me more about your child’s health from birth until two years; (9) tell me more about your child’s nutrition from birth until two years; (10) describe your relationship with your child from birth until two years; (11) how did you play with your child from birth until two years?; and (12) describe any challenges regarding your child since the time he/she was born until two years of age. All interviews were audio-recorded and transcribed verbatim, as preparation for the data analysis. 

### 2.5. Data Analysis

**Figure 1:**
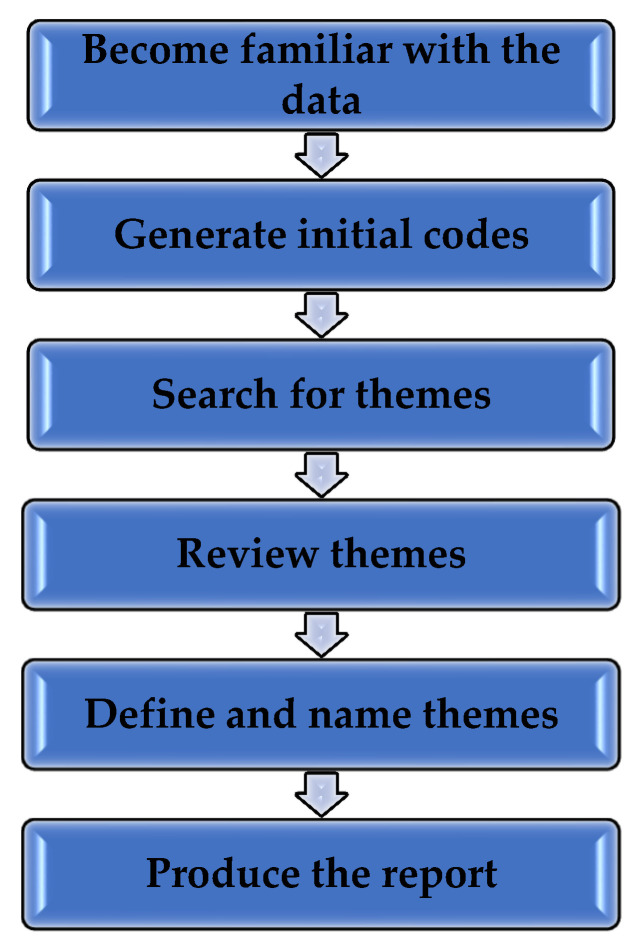
Phases of thematic analysis.

Thematic analysis was utilized to analyze the qualitative data, in order to identify various themes [46]. This involved six phases [47,48], as seen in Figure 1. Additionally, QDA Miner Lite, a free version of the Computer-Aided Qualitative Data Analysis Software (CAQDAS), version 1.4, Provalis Research, Montreal, QC, Canada, was used to assist with the data analysis process [49]. 

## 3. Results

The results were based on interviews with 18 biological mothers of absent learners in the Foundation Phase. The biographical data of the participants are presented in Table 1. 

**Figure 2:**
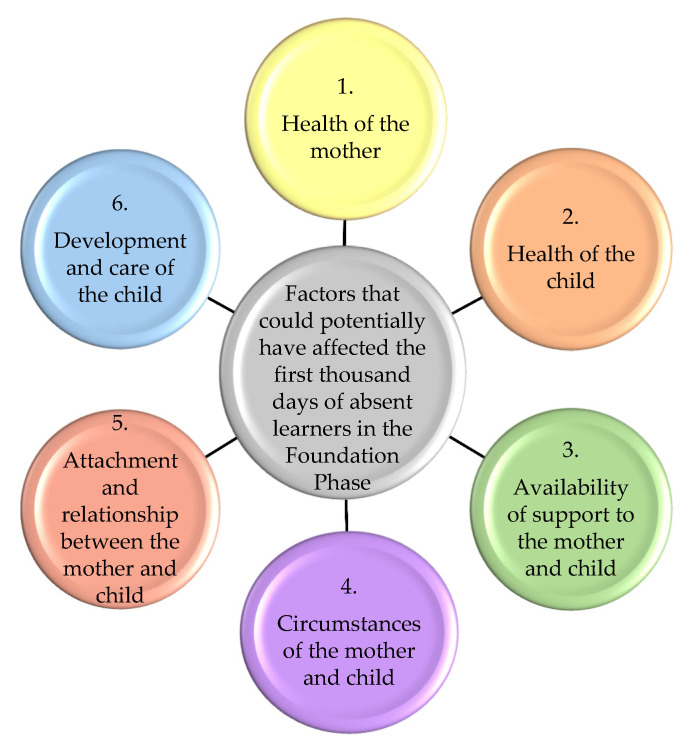
A graphical representation of the six main themes (condensed version) that could potentially have affected the Figure 1000. days of absent learners in the Foundation Phase.

The data analysis revealed six themes, which were further divided into sub-themes. The sub-themes indicated the various factors that could potentially have affected the first 1000 days of the absent learners in the Foundation Phase. See Figure 2. 

### 3.1. Theme 1: Health of the Mothers during the First 1000 Days of Their Children’s Lives

This theme described the physical and mental well-being of the mothers, and other factors that played a role in their overall health, such as nutrition and substance use/abuse.

#### 3.1.1. Sub-Theme 1.1: Physical Health of the Mothers

Approximately half of the participants indicated having good physical health during their pregnancies, while the rest of the participants recalled some health issues, such as heartburn, morning sickness, high blood pressure, and tuberculosis (TB). Many of participants experienced complications, especially during the birth process, for example, participant 18 shared: “… but when I went into labor with her, then with the push, then the umbilical cord was stuck around her neck”.

#### 3.1.2. Sub-Theme 1.2: Mental Health and Emotional Well-Being of Mothers

Although many participants described their pregnancy experience in a positive light, some defined their pregnancy as a negative experience. The participants expressed a variety of stressors (external, internal, or both). Some participants were still teenagers when they got pregnant, which contributed to excessive stress: “I was still in school … and I did not know what do to with the child” (Participant 16). Many participants indicated their current circumstances as stressful and overwhelming, such as the relationship with their child’s father or intimate partner: “Oh, he hit me that evening. He took the belt. He pulled my hair … My stomach was blue from how he hit me” (Participant 17).

Some participants also experienced internal stressors, as expressed: “I felt like a disappointment, felt like I’ve disappointed them [family] with the pregnancy” (Participant 2). Various participants experienced the birth process itself as a strain: “… it is a fear in you, because if you make a mistake here, then it is your fault. Then I panicked …” (Participant 18). The poor mental health of Participant 17 was further portrayed by serious indicators of attempts to harm her child: “I cannot anymore. I left the child at the hospital … I walked out without the child. I do not want the child … I am tired of being a mother …”. On another occasion, the same participant tried to kill the baby: “I told myself, throw the child in the dam of water … we are hungry. We don’t have food”. 

#### 3.1.3. Sub-Theme 1.3: Nutrition of Mothers

Numerous participants felt they ate healthy during their pregnancy, whereas some participants admitted maintaining poor nutrition: “I ate a lot of junk food … I did not worry about what I was eating” (Participant 4). Many participants experienced various challenges regarding their own nutrition, such as morning sickness: “And all that I could force down was water and potato chips. It was only that which I could get in, up to seven months” (Participant 2). Several participants implied a lack of food or limited food as a major challenge: “Food was scarce. There were places [soup kitchens] that handed out food and then I would go and fetch some” (Participant 8).

#### 3.1.4. Sub-Theme 1.4: Substance Use/Abuse of Mothers during Pregnancy

Many participants consumed alcohol, cigarettes, or illicit drugs during their pregnancies, for example, Participant 14 stated: “… about five liters … I just drank a lot”. Only a small number of participants declared a total lack of any substance use during pregnancy. More than half of the participants revealed that they smoked cigarettes during their pregnancies, as stated by Participant 11: “I smoked a lot of cigarettes. Smoked cigarettes the whole day”. A few participants acknowledged using drugs during their pregnancy, which included dagga (Marijuana), Mandrax, or Methamphetamine, referred to as Tik in South Africa. Although very few participants verbalized an understanding of the harmful effects of their own substance abuse on their child, Participant 17 acknowledged: “Tik and Mandrax, that is why the child was so sickly. And at school too. It is almost as if the drugs left something behind. This is why she takes so long, struggle to think”. 

### 3.2. Theme 2: The Health of Children during the First 1000 Days of Life

The participants shared information regarding their child’s health, and the challenges experienced due to illnesses or hospitalization. This theme further elaborated on the children’s nutrition from birth to two years.

#### 3.2.1. Sub-Theme 2.1: Physical Health of the Children

Many participants mentioned a diversity of illnesses that their child suffered from, including an eye infection, jaundice, gastroenteritis (gastro), respiratory difficulties, kidney disease, colon problems, and TB, some resulting in hospitalization. Four participants who smoked cigarettes while pregnant, revealed that their child endured various types of respiratory difficulties after birth, as described: “… then there is a wheezing in her chest” (Participant 8). Two participants’ children contracted TB before the age of two years. Information about medical check-ups and immunizations also emerged during the interviews. Certain participants confirmed taking their children for their scheduled medical check-ups and immunizations. In addition, numerous participants experienced challenges as a result of their children’s illnesses, such as transport problems, lack of communication by medical staff, or stress due to the hospitalization of their children. 

#### 3.2.2. Sub-Theme 2.2: Nutrition of Children

Approximately half of the participants breastfed their child from birth, whereas others fed their baby formula milk. Almost half of the participants experienced challenges with regard to breastfeeding, such as not providing an adequate supply of milk, the baby refusing to take the breast, going back to work, and receiving additional TB treatment to prevent the baby from contracting TB through breastfeeding. 

The participants indicated various ages when their child started to eat solid foods, including from as young as one month to approximately a year. Several participants reported that their child ate healthy food, whereas some participants listed unhealthy food items, such as “Sweets, very much so” (Participant 4). When some of the children in this study were introduced to solid foods, they encountered various challenges, as described by Participant 14: “There was little food. I had to walk with her [child], looking where we could eat”. Several participants referred to their child’s weight as healthy and normal, although three of the participants commented on their children being underweight or overweight, as quoted by Participant 11: “She was a little bit overweight”. 

### 3.3. Theme 3: Availability of Support to the Mothers and Their Children during the First 1000 Days of the Children’s Lives

The participants discussed their experience pertaining to the support they received. They mentioned receiving good to poor support from different groups of people. Furthermore, the participants also mentioned challenges in their relationship with these groups of people, and their child’s relationship with them. 

#### 3.3.1. Sub-Theme 3.1: The Mothers’ Experience Regarding Support

Several participants were unmarried and living with their families when they found out that they were pregnant, which impacted the response from others and the support they received. Participant 16 experienced antagonism from her strict father and shared: “He wanted to put me out [out of the home]”. A few participants faced negative responses from their child’s father: “He told me it was not his child. … he degraded me” (Participant 2), whereas two participants revealed positive responses from their children’s father. Furthermore, some participants had a positive experience regarding support that contributed to optimistic feelings, as stated by Participant 16: “He [friend] made me feel that there was hope”. Some participants had negative experiences regarding support, as indicated: “It is difficult if you are pregnant and you don’t have good support” (Participant 12). 

#### 3.3.2. Sub-Theme 3.2: Support from the Children’s Biological Fathers

During pregnancy, several participants felt supported by the biological father: “Oh, if my feet were swollen, he massaged it. He was there … if I had my date at the hospital, he came along … he was always there” (Participant 8). However, some participants did not feel supported by their child’s father: “He did not help me. He was only there because he had to, but he never helped me like other dads helped their wives or girlfriends” (Participant 13). A few participants highlighted the practical assistance of the father: “Then he cleans up, and he puts on the rice and peels the potatoes. He helped me with everything” (Participant 7). For one participant, the support was mainly financial, whereas Participant 9 also received personal involvement. 

Several participants conveyed challenges pertaining to their relationship with their child’s biological father, which included unstable relationships, break-ups, conflict, substance abuse, a psychiatric disorder, physical abuse, criminal activities, distrust, and financial challenges. 

Five of the participants revealed that their children had a good relationship with their biological father. However, several participants described a poor or absent relationship between their child and the biological father, as specified by Participant 5: “But he was still with her at three months and then he did not come again … when she was two years old, he came again, but then she did not want anything to do with him …”. 

#### 3.3.3. Sub-Theme 3.3: Support from Family Members and Others

Most participants identified family members, especially their own biological mothers who supported them during their pregnancy. The support of one participant’s mother influenced her thoughts on considering an abortion: “Then my mom said, ‘No, abortion is not an option’. She would help me” (Participant 2). Beside their mothers, the participants also mentioned the support received from other family members or other people supporting them, such as neighbors, friends, and colleagues. For one participant, her brother’s support made a big difference in her life: “My brother came down [to the hospital] and brought me stuff … and I knew that he would help me” (Participant 16). However, a few participants reported a total lack of support from others: “Although my mom was there, her cousins, aunts … they did not help me. I went through everything on my own” (Participant 13).

Referring to the child’s relationships with other people, numerous participants elaborated on the integral role family members like grandparents and siblings played in the lives of their child: “He was really spoiled by his sisters. They dressed him up … they were crazy about him” (Participant 7). 

### 3.4. Theme 4: Circumstances of the Mothers and Their Children during the First 1000 Days of Their Children’s Lives

The participants shared information pertaining to their circumstances during their child’s first 1000 days that included living and financial circumstances.

#### 3.4.1. Sub-Theme 4.1: Living Circumstances

The type of housing varied and included houses, flats, and even informal structures made from wood or corrugated iron. More than half of the participants reported positive aspects regarding their living circumstances during their pregnancy. On the contrary, many participants lived in informal structures without water or electricity. They experienced various challenges with regard to their living circumstances. Participant 8 lived with the family of her child’s father, but the house was overcrowded with 20 occupants in a small house with no electricity. Other challenges mentioned were substance abuse within the home, as stated by Participant 16: “My mom drank. My dad was on Mandrax”. 

It appears that some participants’ living circumstances improved in the time-period after their child’s birth. Participant 18 relocated to another home after her child’s birth: “It was nice. The structure was small, but is was warm, comfortable and not leaking or so. It was quite okay”. Furthermore, several participants expressed increased conflict after the arrival of the baby: “And in the home, my sisters and I had a lot of fighting. The child cried, they couldn’t sleep … and then the other grandchildren felt now they were being neglected …” (Participant 16).

#### 3.4.2. Sub-Theme 4.2: Financial Circumstances

Most participants indicated the nature of their employment, along with the number of people working in the household. Several participants gave an account of favorable financial circumstances: “He [child’s father] also had a full-time job. Financially, it went well during my pregnancy. His income was enough” (Participant 7). However, numerous participants experienced financial strain, as they themselves or their family members only worked part-time: “It was very difficult. My father-in-law did char [part time] jobs … and my mother-in-law also did char [part time] jobs, but only two times a week or once a week …” (Participant 8). Some participants were teenagers, and left school when they got pregnant: “Like me, I do not have experience of work and so. Grade 10 out of the school” (Participant 12). 

Some participants found employment after the birth of their child that relieved the financial strain: “… I went working. And then it started to go better” (Participant 16). In contrast, numerous participants experienced severe financial difficulty, and were consequently not able to sufficiently provide for their own needs and the needs of their baby, as expressed by Participant 10: “Then there were those Fridays when we did not even have money for a box of milk for the child. We were not in a financial position to give the child what was due to her”. 

### 3.5. Theme 5: Attachment and Relationship between the Mothers and Their Children during the First 1000 Days of Their Children’s Lives

The participants discussed their attachment to and relationship between themselves and their child, as seen in the following sub-themes. 

#### 3.5.1. Sub-Theme 5.1: Attachment and Relationship between the Mothers and Their Children during Pregnancy

The participants shared their initial responses when they found out they were pregnant: “I was happy” (Participant 5), and “I was thrilled. I was very glad” (Participant 10). More than half of the participants experienced various negative emotions: “First, I panicked. Thought … ‘I already have a child and circumstances are not favorable” (Participant 18). This study revealed the strong gender preference of many of the participants, and their emotions regarding gender: “Excited, and the day I found out it was a girl, then I was even more happy” (Participant 6), whereas Participant 12 stated: “When I found out it was a girl it was too late already … I was actually very angry”. 

Several participants considered abortion or adoption: “In the first place, I wanted an abortion …” (Participant 4), and Participant 17 shared: “I told myself, ‘I am going to let the child be adopted by other people’”. 

Most participants expressed positive thoughts and emotions towards their unborn baby when they felt the movement of the baby: “I was very happy about the kicks. And to see on the sonar how he was moving and carrying on. It was beautiful. It was nice” (Participant 4), and “I’ve bonded with him while I was pregnant” (Participant 2). Additionally, many participants described how they interacted with their unborn child: “If I was alone with him and he kicked me, then I talked to him. And I listened to music, always with the phone, then I put the phone next to me …” (Participant 2). 

#### 3.5.2. Sub-Theme 5.2: Attachment and Relationship between the Mothers and Their Children from Birth to Two Years

The participants described their initial response towards their baby after birth: “I was in love with him from day one. That moment when they put him in my arms, I was in love with the child, until now” (Participant 7). Some participants recalled their immediate interactions, as voiced: “When she was lying there after her birth, I played with her or sang for her” (Participant 1). For two participants, the birth of their children created a sense of responsibility and motivation: “Then after that [the child’s birth], then I thought to myself, ‘I must stand out a bit’ and I did stand out for her” (Participant 14).

During the time-period from birth up to two years, many participants expressed their positive responses: “You have that bond with your child” (Participant 8) and “I was very attached to her” (Participant 9). However, some participants experienced negative emotions or thoughts at times: “I don’t feel up to baby today” (Participant 11), and “… can’t the child die or something, throw the child away” (Participant 17). 

Numerous participants mentioned that their child constantly wanted to be with them with regard to attachment and the relationship from their child’s side: “If I am not with her, then she cries. She was very close to me. I could not walk away … she looked for me” (Participant 1). Two participants distinctively described their children’s positive responses and attachment towards them: “And she always clung, grabbed and said, ‘Mommy’, then grabbed me and held onto me” (Participant 8).

### 3.6. Theme 6: Development and Care of the Children during the First 1000 Days of Their Lives

This theme described aspects relating to the children’s development, the care, and protection the participants provided to their child, as well as the way the participants stimulated and played with their child. 

#### 3.6.1. Sub-Theme 6.1: The Children’s Development

The participants mentioned various aspects pertaining to their child’s physical development, such as hearing, movement, rolling, and crawling: “We wanted to see if she could hear correctly, if she could listen” (Participant 9). Some participants referred to their child’s emotional needs, whilst some participants showed insight into their child’s linguistic and cognitive development: “She wanted to know about everything. She was at that age where she walked, she experimented with things” (Participant 11).

Relating to their children’s milestones, some participants indicated that their child reached some milestones early: “She did not crawl for long, then she started to stand up against things and began to walk. She developed quickly” (Participant 12). A few participants felt their child failed to meet some age-appropriate milestones or had developmental delays: “She does not catch or understand so well what I say. And the one eye is here while the other eye turns that side” (Participant 17). 

#### 3.6.2. Sub-Theme 6.2: The Mothers’ Care and Protection of the Children

Participants referred to different aspects of caregiving, and many shared examples of how they ensured good caregiving: “So, I must make sure that he has everything, because the nanny must be paid, she must be looked after, her food must be ready—what she eats, her milk, clothing, everything” (Participant 3). Three participants explicitly noted that their children had all they needed, referring specifically to their physical needs: “Provision was always made for him … he never lacked anything. He was never hungry or so” (Participant 2). 

Some participants also indicated that emotional caregiving was important, as stated by Participant 14: “I gave her love”. Three participants showed an awareness of risks, and the need to protect their children: “She can maybe just be in the yard at the back, then I would look for her. Don’t let her play in the street. Scared the cars will drive over her” (Participant 1). 

Furthermore, the participants revealed a range of challenges regarding the caregiving of their children: “Sometimes yes, he was tired, but he always battled to sleep … when he became older, it was always a struggle to get him to sleep” (Participants 2). Two participants experienced feelings of inadequacy: “… didn’t know what to do …” (Participant 15). For some participants, their physical or emotional well-being influenced their caregiving: “I think it was the cut [cesarean] that caught me off guard … I had a lot of pain, so I did not have time for her. So, I neglected her a little bit …” (Participant 9). An additional challenge for many participants was the lack of baby goods, due to financial strain: “Sometimes, when there was no cereal and I didn’t have money, then the child cried the whole day and so. When there were no [nappies]” (Participant 17).

#### 3.6.3. Sub-Theme 6.3: Stimulation and Play

This study revealed different ways in which the participants incorporated stimulation and play into the various stages of their children’s lives. During infancy and the first few months, the participants reported the following: “… tickled him on his chest” (Participant 3), “… sing for them, then swing them a little bit” (Participant 5), and “… played hide and seek” (Participant 13). Two participants hanged items in the air for their children to play with: “Then I maybe make the balloons … with a string to just where his hands could touch, then he plays with it” (Participant 7). 

Some participants played with their children when they became older: “I played with him with cars” (Participant 2), or other activities, such as “sang for her, danced” (Participant 5). Some mothers used specific activities to intentionally stimulate and educate their children, such as walking in the garden, or using items in their home to teach them about shapes and colors. However, two participants did not regard it as necessary to play directly with their children, as they felt that their children could play with their siblings, as captured: “No, because most of the time she played with her sisters. So, I did not really play like that, like in play with her …” (Participant 6). Two participants mentioned their child’s father playing with the child, as explained by Participant 18: “… and then I would go and call the father … and then we [participant, child and father] played together”. 

## 4. Discussion

### 4.1. Discussion of Findings

The data provided insight into the various factors that could potentially influence the first 1000 days of the lives of children. In the context of this study, these factors relate to the three key areas outlined in the First 1000 Days campaign that was launched in the Western Cape, South Africa, namely (1) health and nutrition; (2) love and attention; and (3) play and stimulation [10].

The health of mothers (Theme 1) involved their physical and mental health, nutrition, and substance abuse of the participants, which played a vital role during their pregnancy and after the birth of their children. Many participants in this study suffered from severe illnesses and medical conditions, such as extreme high blood pressure, a stroke, and TB during their pregnancy. These conditions increased the risk of complications for both the mothers and their children [50,51,52]. Numerous complications occurred during pregnancy, especially during the birth process, including the following—preterm birth, low birth weight, umbilical cord prolapse, ectopic pregnancy, hypertension, and breech births [53]. In addition, various children in this study suffered from oxygen deprivation after birth, which could have caused disabilities and developmental delays [54,55]. The participants revealed various aspects that influenced their emotional and mental well-being during this time-period. Although some participants encountered positive experiences and emotions during their pregnancy, others endured severe stress, especially since it seemed that all these pregnancies were unplanned. Several participants described their pregnancy experience in a negative light, as a result of morning sickness, swelling, uncertainty, sleeping challenges, and high blood pressure caused by their pregnancy. A mother’s well-being during pregnancy influences the baby’s well-being [56], which could have long-lasting physical, cognitive, and emotional outcomes for the child [57]. During pregnancy, the participants experienced stressors related to their circumstances. Some participants were teenagers during their pregnancy, and they had to attend school, and the relationship with the biological father also added strain. Numerous participants experienced internal stressors, especially relating to the announcement of the pregnancy to their family, which caused intense emotions. The findings also indicated that a few participants experienced sadness, anxiety, guilt, and feelings of worthlessness, which could possibly be associated with symptoms of depression [58]. Prenatal exposure to maternal depression could affect the brain development of the fetus [59].

During the time from the child’s birth to two years, the stressors mentioned were mostly related to motherhood and losing a loved one. One of the mother’s mental health after the birth of her child showed signs of postpartum depression [60,61], or even postpartum psychosis [62], as she attempted to kill her baby. Although most participants ate healthy during their pregnancy, poor nutrition occurred amongst some participants. Nutritional deficiencies during pregnancy could affect children’s cognition, behavior, and productivity later in life [63,64]. The occurrence of substance use/abuse during pregnancy was considerably high, with cigarette smoking the highest, followed by alcohol use, and lastly, drug abuse. Illicit drugs that were consumed during pregnancy included Methamphetamine (Tik), dagga (Marijuana), and Mandrax. Substance use/abuse during pregnancy poses severe risks to both mothers and their unborn babies, especially physical and neurological damage [65]. Prenatal use of alcohol, as consumed by many participants, could have long-term consequences on their children’s cognitive, behavioral, social, and emotional development [66]. The prenatal use of alcohol by the participants in this study corresponds to the high prevalence rates of FASD in South Africa [67]. 

The health of the children (Theme 2) during the first 1000 days establishes the foundation for optimal health and development later in life [68]. Although a few participants highlighted their children’s good health, many of these children in this study experienced poor health. Several children suffered from oxygen deprivation directly after birth, which can cause disabilities, developmental delays, and other long-term effects [55,56]. The children of four participants who smoked during their pregnancy experienced respiratory problems after birth, which could possibly be caused by prenatal cigarette smoking [69]. A number of children of participants suffered more severe illnesses and conditions, such as TB, kidney disease, colon problems, and physical disabilities. A few children were hospitalized, causing sleep deprivation and severe psychological stress. A number of participants confirmed taking their children for their medical check-ups and immunization at the scheduled times, which played a vital role in the health of their children [70]. The study also focused on the children’s feeding and nutrition, from birth to two years, and many participants breastfed their babies during the first few months, as recommended by the World Health Organization [71]. Some participants complied with the guidelines set by the Department of Health in South Africa, by introducing solid foods to their infant’s diet at six months [72]. Many participants provided good food choices like fruit, vegetables, meat, and dairy products, as suggested by the Australian Government [73]. In terms of the children’s weight, several participants regarded their children’s weight as healthy, while a few stated that their children were underweight or overweight. Many participants experienced challenges regarding their children’s feeding and nutrition, with a lack of or limited food. Nutritional deficiencies occurred, due to poor nutrition and other challenges, which possibly caused stunting in some of these children, which affected their brain development and resulted in learning difficulties at school [74]. 

Support (Theme 3) from others could influence the health and well-being of individuals [75]. The participants’ experience of support during pregnancy was affected by their emotions and thoughts, as well as the announcement of their pregnancy and other people’s responses. Many participants were unmarried at the time when they became pregnant, and the announcement of the pregnancy caused anxiety and fear. Several mothers experienced family members not talking to them, threatening to put them out of the house, or the biological father denying fatherhood. Many participants felt supported during their pregnancy and after the birth of their child, while numerous participants experienced a lack of support that resulted in a sense of loneliness. A lack of support caused the participants to be more vulnerable to depression and anxiety. Support from the biological father is paramount [76], as experienced by some participants. A number of participants received support from the child’s father, however, several participants experienced no support from the biological father during that time-period. Many participants expressed challenges pertaining to their relationship with the biological father, including an unstable relationship, conflict, drug abuse of the father, and lack of financial support. A few biological fathers engaged with their children, and played an active role in their lives, which could have had a positive influence on these children’s social, emotional, and cognitive development [77]. It was clear that other significant people, especially the grandparents, siblings, and the participants’ male friends, played a positive role in the lives of their children.

The circumstances of the mothers and their children (Theme 4) referred to their living and financial circumstances during pregnancy and birth to two years. More than half of the mothers described their living circumstances as suitable and pleasant, while others experienced adverse living conditions, such as unsuitable housing, lack of water or electricity, a significant amount of family conflict, and substance abuse in the home, which led to an increased vulnerability experienced by the mothers and their children [78]. The living circumstances remained the same in most cases after the birth of the child, however, there were changes and even improvements for some participants during that time-period. Financial circumstances had a huge impact on the participants, as most of them experienced some form of financial strain, often lacking the resources to supply their child’s basic needs.

The attachment and relationship between the participants and their children (Theme 5) during pregnancy, and during the two-year period after birth were influenced by various aspects. Some participants were happy and excited when they found out they were pregnant, whereas many of them initially experienced negative emotions, such as shock, confusion, disappointment, anger, sadness, and panic, due to an unplanned pregnancy [79]. These negative emotions made the mothers more susceptible to the risk of maternal depression [80]. The child’s gender influenced the emotional responses of participants. Many participants felt relief, happiness, and excitement when the gender coincided with their preferences. Several participants considered abortion or adoption. The movement of the unborn baby activated various positive emotions, such as happiness, excitement, enjoyment, love, acceptance, and protection. These positive emotions indicated signs of bonding [81,82]. Some participants interacted with their unborn babies in various ways, by talking and singing to them. These interactions promoted the children’s development [81], and enhanced bonding between them and their unborn babies [83]. Many participants expressed positive initial responses, interaction, and emotions, directly after the birth of their child, while some experienced an internal motivation for self-improvement. These positive responses immediately after birth influenced them to be more inclined towards their children’s needs [84]. It was noticeable that attachment between the participants and their children formed during the two-year period after they were born. However, a few participants expressed certain negative emotions and thoughts towards their child, which included irritation, lack of attachment, and serious harmful emotions and thoughts by one of the participants specifically. 

The development and care of the children (Theme 6) involved a variety of aspects. Although most participants showed an understanding of some of their children’s developmental needs—especially their physical needs—many participants had a limited understanding about their children’s emotional, linguistic, and cognitive developmental needs. Some participants felt that their children met their developmental milestones, while others highlighted their children’s rapid development. One of the mothers who abused drugs during her pregnancy, emphasized her child’s cognitive and physical developmental challenges. Many participants used ‘serve and return’ to stimulate and play with their babies, which is beneficial for building a strong foundation in the child’s brain architecture [85]. Many participants used educational toys or activities to incorporate stimulation and play into their children’s lives. Only some participants played with their children when they were older, since they felt that the children could play with their siblings.

Reflecting on the findings of this study and various theories relating to child development, it appears that these theories play a significant role in the following way. The first two stages of Erikson’s Psychosocial theory [86,87] refer to children’s development of trust and autonomy. The sensorimotor stage of Piaget’s theory of cognitive development was noticeable in the way the participants described their children’s understanding of their environment through different activities involving their senses. Attachment between the participants and their children, as indicated by the attachment theory [28], was highlighted as one of the themes and occurred various times in this study. The four phases of the attachment theory [28,86] were also evident during the time from birth to two years, as the participants described the bond between them and their child, and the reaction of their child when separation occurred. However, specific types of attachment [28,86] were not definite. Bronfenbrenner’s bioecological theory [88] was clearly evident throughout the study, where both the mothers’ and children’s interaction with various systems played a vital role. Lastly, Maslow’s hierarchy of needs [87,89], mainly the first three needs, namely (1) physiological needs; (2) the need for safety; and (3) the need for love; were strongly visible in the findings. A lack in basic physiological needs, such as food and baby goods due to poverty, had an enormous effect on some of the participants, and were highlighted by many of them.

Finally, it should be noted that although the aim of this study was not to determine a correlation between the first 1000 days and absenteeism, the findings of this study indicated that many factors played a vital role during the first 1000 days of the absent learners in this study. Literature further confirmed that specific aspects revealed in the findings of this study, such as nutritional deficiency [25], substance abuse during pregnancy [29,30], toxic stress [32], attachment, [34] and stimulation [36] could have negative effects on the schooling of children. Some of these aspects include academic, social, emotional problems at school, and school absenteeism. 

### 4.2. Limitations

This study involved participants from a rural community in South Africa, characterized by high rates of poverty. The findings might, therefore, not be representative of all children in other communities, pertaining to their first 1000 days of life. Conducting the study on a small scale with a total of 18 female participants from one area limits the generalizability of the findings in terms of being representative of all children’s first 1000 days. The interview schedule that was utilized during the interviews was limited to 12 questions, resulting in some areas not being explored. For example, the age of the mothers during pregnancy, or whether the pregnancy was planned were not specifically asked. Another aspect that was not explored, was the mothers’ substance use/abuse after birth while they were breastfeeding, since this was not a definite question, and not mentioned by the mothers. 

### 4.3. Implications for Future Research

Future research should include a larger sample size, or be conducted in other communities besides rural communities. Future qualitative studies should explore other aspects that could have affected the first 1000 days of the lives of children, for example, whether mothers consumed alcohol or used other substances while breastfeeding. Since the first 1000 days are affected by various factors, a quantitative study could provide information with a numerical value relating to these different factors. Future research should evaluate the impact of numerous first 1000 days initiatives worldwide, to provide insight and recommendations regarding the best interventions and strategies. 

### 4.4. Contributions to Communities and Professionals Working in the Field of Early Child Development

This study makes a contribution by increasing our understanding of the various factors that could potentially have affected the first 1000 days of absent learners in the Foundation Phase. These insights could provide Government, NGOs, communities, and professionals working in the field of Early Childhood Development (ECD) with recommendations that could be considered during their planning of future programs and interventions. These recommendations include—(1) educate the broader communities on the importance of the first 1000 days through continual social media campaigns, by making use of platforms like the radio, television, newspapers, and Facebook; (2) incorporate the guidelines set out by the World Health Organization on the identification and management of substance abuse during every antenatal visit, to screen the past and present substance use/abuse of mothers; (3) provide support groups for pregnant mothers and mothers with children up to two years, in order to offer emotional support, education, and practical guidance regarding nurturing care, attachment, and stimulation of children in this time-period; (4) offer workshops and interactive events to biological fathers and extended family members to establish meaningful support to mothers during pregnancy and after their child’s birth; (5) add a module that focuses on the first 1000 days to the tertiary training curriculum of social workers, healthcare workers, ECD practitioners and educators; and (6) include first 1000 days initiatives in national and international policies, to compel and guide countries regarding the implementation of these interventions during this critical time-period. 

## 5. Conclusions

Research on a South African and international level highlights the many factors that could influence the first 1000 days of life—between pregnancy and the second birthday of children. Whilst research emphasizes that adequate nutrition and care during this unique time-period determine whether children will thrive and reach their full potential, it is evident that this is not always true for all children. Based on the findings of this study, it was concluded that a substantial number of factors, such as the health and nutrition of both the mothers and their children, substance use/abuse during pregnancy, toxic stress, support to the mothers and their children, attachment, attentive care, stimulation and play could have played a role during the first 1000 days of the absent learners in this study. Most of these factors relate to the Western Cape Government’s First 1000 Days campaign launched in South Africa, entitled ‘Right Start Bright Future’. It was confirmed by literature that the factors relating to the First 1000 Days campaign could potentially have affected the development of children during their first 1000 days, with a lifelong impact on, amongst others, their schooling.

In addition, it is imperative that support is offered to both mothers and their children during this critical time. It is, therefore, essential that NGOs, practitioners, and even the private sector are educated on the importance of the first 1000 days of life, in order to motivate them to collaborate with government departments to strengthen the support of mothers and children. Greater collaboration and multi-sector partnerships are, therefore, needed to improve services focusing on the first 1000 days of life, enabling all children to thrive and transform.

## Figures and Tables

**Table 1:** Biographical data of participants.

Participant	Current Age during the Study	Current Relational Status during the Study	Total Number of Children	Position of Absent Learner in Relation to Other Siblings
Participant 1	31 years	Single	3	Eldest
Participant 2	28 years	Married	3	Eldest
Participant 3	40 years	Married	6	Fourth child
Participant 4	31 years	Single	2	Youngest
Participant 5	26 years	Single	3	Eldest
Participant 6	37 years	Long-term relationship	7	Fifth child
Participant 7	42 years	Married	5	Youngest
Participant 8	39 years	Married	7	Fourth child
Participant 9	31 years	Married	3	Eldest
Participant 10	44 years	Long-term relationship	5	Youngest
Participant 11	37 years	Divorced	4	Third child
Participant 12	29 years	Single	2	Youngest
Participant 13	48 years	Single	4	Youngest
Participant 14	33 years	Married	2	Youngest
Participant 15	28 years	Long-term relationship	2	Eldest
Participant 16	29 years	Long-term relationship	2	Eldest
Participant 17	32 years	Long-term relationship	2	Youngest
Participant 18	34 years	Long-term relationship	3	Middle child

## Data Availability

The data presented in this study are available on request from the corresponding author. The data are not publicly available due to privacy.
